# The long and winding road: On the endoplasmic reticulum of *Plasmodium* and implications for pathogenesis

**DOI:** 10.1371/journal.ppat.1014539

**Published:** 2026-08-25

**Authors:** Antonia Blank, Solomon Ngwira, Manuel A. Fierro

**Affiliations:** 1 Department of Genetics and Biochemistry, Clemson University, Clemson, South Carolina, United States of America; 2 Eukaryotic Pathogens Innovation Center, Clemson University, Clemson, South Carolina, United States of America; Bernhard-Nocht-Institute for Tropical Medicine, GERMANY

## Abstract

As a eukaryote, *Plasmodium*, the causative agent of malaria, is reliant on its endoplasmic reticulum (ER) for survival. However, as a protozoan pathogen, *Plasmodium* has also adapted its ER to support its parasitic lifestyle. This Pearl focuses on the interplay between conserved ER functions in *Plasmodium* such as cell signaling, protein synthesis/secretion, lipid production, and cellular stress, and their contribution to parasite pathogenesis. We further summarize the current knowledge about the role of the ER in pathogenic processes such as motility, egress/invasion, host-cell remodeling, and transmission throughout the blood, liver, sexual, and mosquito stages. Collectively, we hope that discussing the *Plasmodium* ER as both a conserved cellular hub and a source of parasite-specific biology offers promising opportunities for antimalarial strategies.

## Introduction

The endoplasmic reticulum (ER) is a eukaryotic organelle that functions as a hub for protein synthesis/trafficking [1,2], calcium (Ca^2+^) signaling [3], and stress responses [4]. Understanding the role of the ER in adaptations like parasitism reveals new insights into anti-pathogen treatment. Towards this goal, this review will focus on the intracellular parasite *Plasmodium*, the causative agent of malaria. *Plasmodium* maintains conserved ER core functions present across other eukaryotes [5–7] including Ca^2+^ storage [8], glycosylation [9], protein quality control [10], and protein secretion [11]. We will discuss the known functions of the *Plasmodium* ER, their impact in parasite pathogenesis, and implications for antimalarial development.

## The architecture of the ER across the *Plasmodium* life cycle

The ER is a dynamic organelle whose architecture is maintained by various proteins [12]. One ER-shaping protein called Yop1, which in other eukaryotes interacts with reticulons to organize ER tubules, is important for ER structure and virulence in *P. berghei* [13]. Other ER-shaping proteins have been identified bioinformatically [14], but their functional conservation across the life cycle remains to be studied. In contrast, the general architecture of the ER across its complex life cycle is well characterized.

### Blood stages

**Fig 1:**
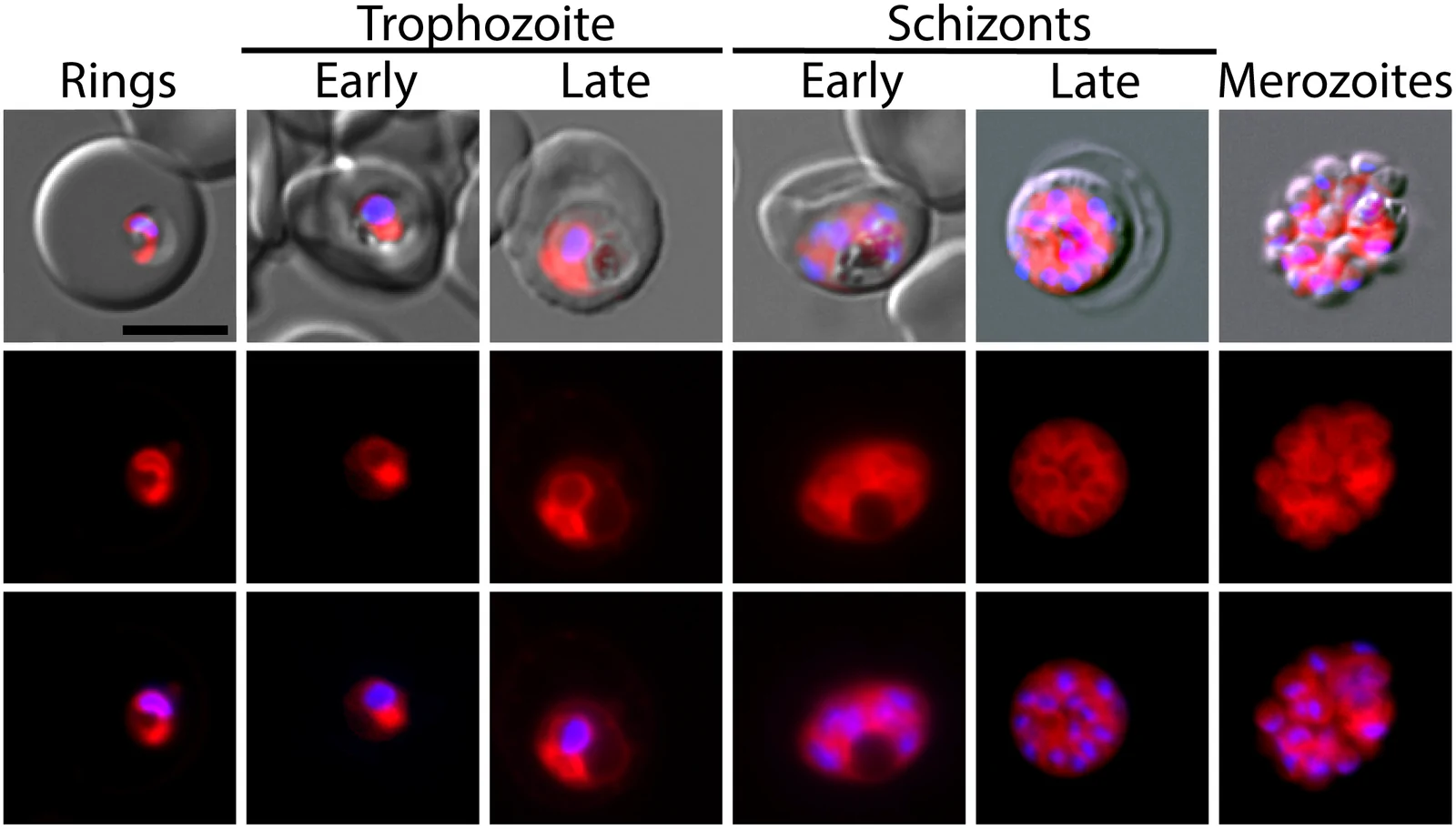
The architecture of the ER during *Plasmodium falciparum*’s blood stages. Representative images showing the structural development of the ER across the blood stages. The ER and nucleus were visualized by staining parasites with ER Tracker (red, Invitrogen) and Hoescht (blue, Invitrogen). Images were taken under the same conditions. Scale bar: 5 µm for all images.

Like other eukaryotes, the ER of *Plasmodium* wraps itself around the nucleus, producing a distinctive peri-nuclear pattern observed in the blood stages of *P. falciparum* [15 ^–^ 17] (Fig 1) and *P. berghei* [18]. This peri-nuclear pattern changes from a condensed form with some protrusions in the ring stages, to a larger form that contains several protrusions or condensed foci during the trophozoite stage [15–18] (Fig 1). These protrusions appear to contact organelles like the mitochondria [16] while expansion microscopy of condensed foci has revealed their composition to be compact ER cisternae [17]. Upon entering schizogony, the ER volume begins to extend throughout the cell body, and several interconnected ringlet patterns appear due to the newly synthesized nuclei that have divided in preparation for daughter-cell formation [15–18]. Once schizogony finishes, the ER ringlets sepBlood stagesarate and encapsulate each new daughter merozoite’s nucleus (Fig 1).

### Sexual stages

Sexual stages, known as gametocytes, are a subset group of the blood stage and have a unique morphology compared to their other blood stage counterparts. Using electron microscopy (EM) techniques to study gametocyte organellar ultrastructure, Evers and colleagues [19] demonstrated that the ER of female gametocytes in *P. falciparum* occupies a larger volume compared to male gametes or immature gametocytes. The authors attribute this discrepancy to increased protein synthesis in female gametocytes compared to their male counterparts. Moreover, their data suggests that the ER of both sexes contains several contact sites including the Golgi, mitochondria, the Inner Membrane Complex, and plasma membrane (PPM).

### Liver stages

During the liver stages, the ER displays a similar yet more dynamic structure compared to its blood stage counterparts which is partly due to the dramatic asexual expansion occurring in hepatocytes [20]. Using confocal microscopy, sub-compartmentalization in the ER of *P. berghei* was observed by overlapping yet distinct ER distributions of the soluble, ER chaperone BiP and the ER membrane protein Sec61β [18]. Like the blood stages, large aggregate structures were also visible throughout liver stage schizonts which were determined by EM to be complexed ER tubule bundles [18]. The liver stage ER was also proven to be a single, interconnected organelle throughout the cycle that segregates into separate organelles at the very end of the liver stages, coinciding with newly formed merozoites. Interestingly, like gametocytes, the ER of liver stages also contains regions where ER-PPM contact sites can be observed by EM [18].

### Mosquito stages

Less is known about the role of the ER in the mosquito stages due to the difficulty of acquiring these forms in the laboratory. However, basic structural studies using confocal microscopy demonstrated that the ER of *P. berghei* ookinetes, the stage emerging after gamete fertilization, surrounds the nucleus and extends across the body of the parasite similar to female gametocytes [18]. As the parasites transition into the oocyst stage, the ER begins to structurally mimic that of liver stages with large ER accumulations observed throughout the developing oocysts, culminating in perinuclear ER forms surrounding the nucleus of individual sporozoites. The similarities between liver schizonts and oocysts are likely due to the hyperactive replication that occurs in these stages. Once sporozoites develop, the ER extends across the whole body of the parasite similar to ookinetes [18].

## Function of ER Ca^2+^ in parasite biology

The *Plasmodium* ER is thought to be the primary compartment for Ca^2+^ signaling/storage. As such, it contains mechanisms for replenishing ER Ca^2+^ [21] and ER Ca^2+^ buffering [22]. However, *Plasmodium* lacks an annotated IP_3_/Ryanodine receptor for signal-dependent ER Ca^2+^ release [21]. One candidate, ICM1, has been shown to be important for this process during parasite egress/invasion in *P. falciparum* [23], yet its localization/function across other blood stages remains unknown. Similarly, the ER Ca^2+^-binding protein ERC plays a role in parasite egress from red blood cells (RBCs) [8]. Indeed, most of what is known about the role of Ca^2+^ in *Plasmodium* spp. is generally related to entering [24] and exiting [25] RBCs, male gamete activation [21], and motility [21]. These events are coordinated by signal-based release of apical organelles and all work in conjunction with other secondary messengers like cGMP, cAMP, and xanthurenic acid. More importantly, disruption of Ca^2+^-related events is lethal to parasites showing that identification of all the players involved in ER Ca^2+^ release/storage provides a rich source of potential antimalarial targets.

## *Plasmodium*’s innovative secretory pathway

*Plasmodium* contains conserved mechanisms for chaperone-mediated protein trafficking [22] and retention of ER proteins [26]. These trafficked proteins are then targeted to either internal (i.e., secretory organelles, apicoplast, etc.) or external compartments (i.e., Parasitophorous Vacuole, RBC) [22] and ER protein trafficking mechanisms are a target of emerging antimalarials [27,28]. One innovative trafficking mechanism in the parasite is the process for sending cargo destined to the apicoplast, a relict plastid acquired from past endosymbiotic events, by duplication of ERAD machinery to help import apicoplast-destined cargo transcribed from the nucleus [29]. Another example is the export of proteins to the RBC. In *Plasmodium*, most exported proteins contain a N-terminal *Plasmodium* Export Element (PEXEL) motif which is a proteolytic cleavage site required for export of this class of proteins [30,31]. Upon ER entry, the PEXEL is cleaved by a distinct Sec61-associated complex containing the aspartic protease Plasmepsin V (PMV), replacing the role of Signal Peptidase [32]. While the reason for a separate translocon is unclear, it likely arose to alleviate trafficking of exported cargo through the canonical Sec6-Signal Peptidase complex by deviating it to distinct machinery [33]. More importantly, PMV is essential for parasite viability and the target of several compounds [34,35].

## The role of the ER in protein modifications

The ER is a well-established site for post-translational modifications of nascent proteins. One of these modifications is the addition of disulfide bonds, which form in the ER’s reducing environment. This is carried out by ER-resident Protein Disulfide Isomerases (PDIs) which, in *P. falciparum*, are encoded by four PDIs [36]. Of these four, PDI8 is the only one confirmed to be essential for parasite growth and works with PDI11, PfJ2, and BiP to properly fold proteins in the ER lumen [36]. Another notable modification in the parasites is protein glycosylation which appears to only occur in the ER in contrast to model eukaryotes that additionally use the Golgi for this process [9,37,38]. The most abundant glycan in *Plasmodium spp* is glycophosphatidylinositol (GPI), an essential component of GPI anchors [37] and used by invasion-related proteins like MSP1 [9] and CSP [39]. Moreover, evidence of *O*-fucosylation, and *C*-mannosylation have also been found in the parasites [9, 37, 38] which play a stabilizing role for CSP and TRAP during mosquito and liver stages [9, 37, 38]. While some studies report the presence of *N*-glycosylation [38], it’s not clear if any proteins are extensively *N*-glycosylated in *Plasmodium*. In general, glycosylation in *Plasmodium* appears to function in stage transmission [40,41], providing an attractive antimalarial strategy.

## Lipid synthesis

Synthesis of lipids required for membrane biogenesis and energy occurs in the eukaryotic smooth ER [42]. Despite the need to scavenge precursor molecules, *P. falciparum* contains the essential ER machinery required for production of these necessary lipids [43]. Not surprisingly, the ER has multiple contact sites with various organelles [16^,^ 19] and the PPM [18,19] to facilitate the transfer of lipids as previously demonstrated for PfPSS in the mitochondria [16] and PfVAP/PfVPS13L1 in the inner membrane complex (IMC) [44]. The ER is also a hub for lipid droplet (LD) formation which are crucial for lipid homeostasis. Studies in *P. falciparum* show that LDs are associated with the ER and required for parasite survival [45], yet the molecular players involved in this process are yet to be determined. Lastly, phosphatidylcholine produced by a host-derived precursor plays a crucial role in sexual differentiation showing another potential avenue for anti-transmission strategies [46].

## ER stress pathways and drug resistance

The ER is a major site of stress surveillance and, thus, contains several mechanisms to deal with cellular stress [4,47,48]. Of the three ER-related stress pathways found in eukaryotes (IRE1-mediated, PERK-mediated, ATF6-mediated), *Plasmodium* is only capable of global translational arrest via the PERK pathway [18,28]. Translational arrest is a key feature in the progression of *P. falciparum*’s lifecycle as eIF2α phosphorylation can be identified in terminal stages such as the schizont stage in RBCs [49] or in sporozoites [50]. Interestingly, activation of this pathway has been shown to be induced in the presence of the frontline antimalarial, artemisinin [49], a process modified in artemisinin-resistance parasites [51]. Thus, inactivation of this pathway has important implications to counter parasite recrudescence after artemisinin treatment to improve its efficacy in resistant parasites.

## Conclusion

While generally conserved ER features exist in *Plasmodium*, these parasites have tweaked their use to uniquely fit their parasitic lifestyle. Thus, with the present state of antimalarial strategies, focus on the *Plasmodium* ER can provide novel points of attack against this deadly parasite. Indeed, recent proteomic studies have highlighted conserved mechanisms as well as novel ER-associated proteins whose functional characterization would be of interest to the wide community [44,52,53]. Moreover, by taking advantage of current advances in genetics, microscopy, and proteomics, future *Plasmodium* studies should continue characterizing: (1) the mechanism for ER structural maintenance; (2) how Ca^2+^ is released from the ER; (3) sites of ER entry/exit; (4) all the molecular players involved in glycosylation; and (5) the process of LD formation. We hope this review re-stimulates attention to this fascinating organelle.
